# *N*-Cyclo­hexyl­tryptamine: freebase, bromide and fumarate

**DOI:** 10.1107/S2056989023006217

**Published:** 2023-07-25

**Authors:** Marilyn Naeem, Alexander N. Le, Barbara E. Bauer, Andrew R. Chadeayne, James A. Golen, David R. Manke

**Affiliations:** a University of Massachusetts Dartmouth, 285 Old Westport Road, North Dartmouth, MA 02747, USA; b CaaMTech, Inc., 58 East Sunset Way, Suite 209, Issaquah, WA 98027, USA; University of Neuchâtel, Switzerland

**Keywords:** crystal structure, tryptamines, indoles, hydrogen bonds

## Abstract

The solid-state structures of *N*-cyclo­hexyl­tryptamine (**I**) {systematic name: *N*-[2-(1*H*-indol-3-yl)eth­yl]cyclo­hexa­namine}, C_16_H_22_N_2_, and two of its salts, *N*-cyclo­hexyl­tryptammonium bromide (**II**) {systematic name: *N*-[2-(1*H*-indol-3-yl)eth­yl]cyclo­hexa­naminium bromide}, C_16_H_23_N_2_^+^·Br^−^, and *N*-cyclo­hexyl­tryptammonium fumarate (**III**) (systematic name: bis­{*N*-[2-(1*H*-indol-3-yl)eth­yl]cyclo­hexa­naminium} (2*E*)-but-2-enedioate), 2C_16_H_23_N_2_^+^·C_4_H_2_O_4_^2−^, were determined by single-crystal X-ray diffraction. The freebase compound forms infinite chains along [010] through N—H⋯N hydrogen bonds. The bromide salt is held together by N—H⋯Br inter­actions in two-dimensional sheets along (001). The fumarate salt is held together in infinite three-dimensional frameworks by N—H⋯O hydrogen bonds.

## Abstract 2

The crystal structure of the freebase of the monoalkyl tryptamine *N*-cyclo­hexyl­tryptamine is presented, along with those of its bromide and fumarate salts.

## 1. Chemical context

Tryptamine, an indole with a 2-amino­ethyl sidechain, is a metabolite of the essential amino acid tryptophan. Tryptamine and its derivatives are an important class of biologically active compounds that are found in almost all organisms on Earth. In humans these compounds play significant roles ranging from the function of the gastrointestinal tract to neurotransmission and control subjective phenomena like happiness. The most abundant of these compounds, occurring naturally in the body, are primary tryptamines like tryptamine itself and serotonin (5-hy­droxy­tryptamine; 5-HT) (Palego *et al.*, 2016 ▸). 
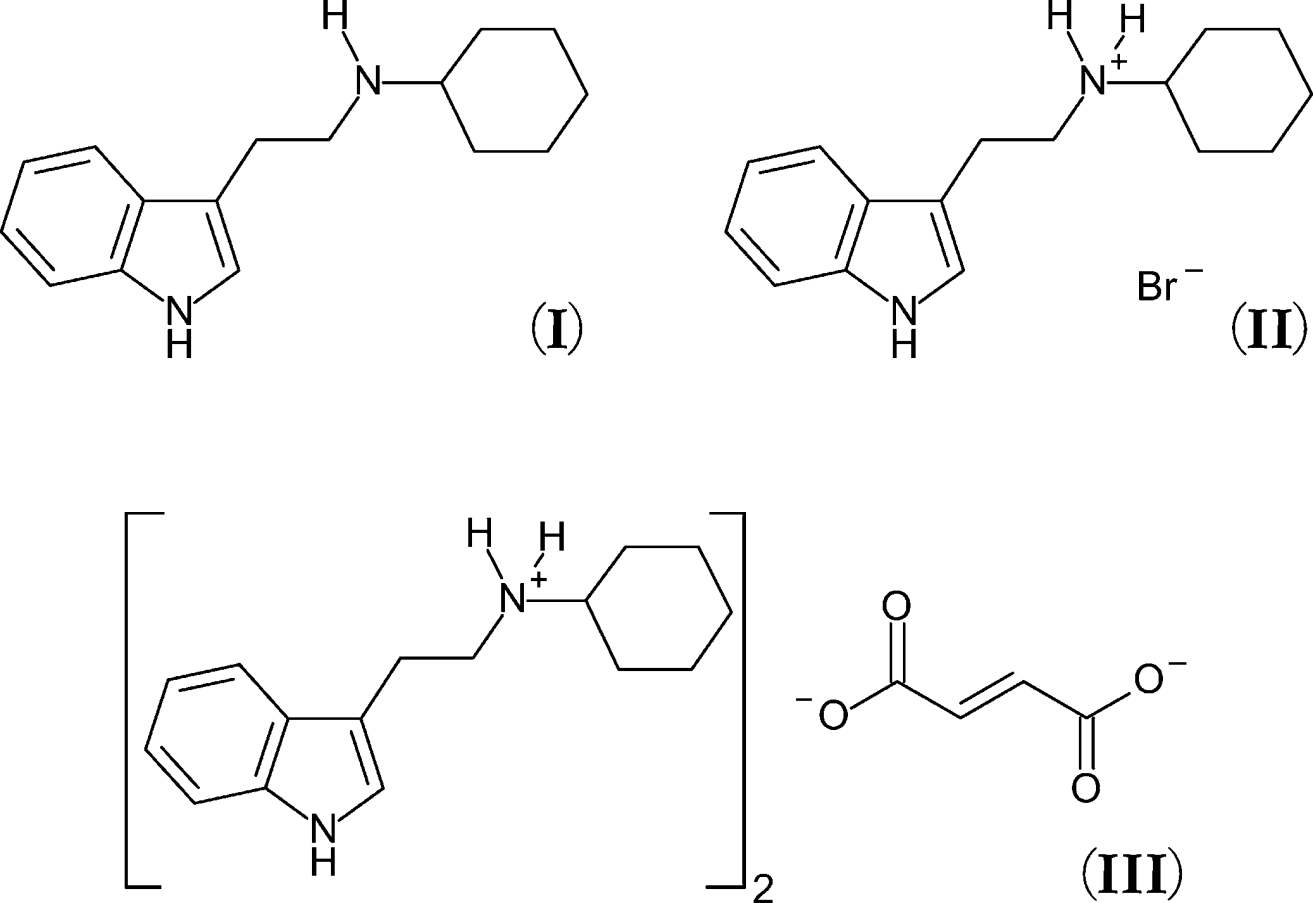


There are many well-known tertiary (dialk­yl) tryptamines, including the natural products *N*,*N*-di­methyl­tryptamine (DMT), 5-meth­oxy-*N*,*N*-di­methyl­tryptamine (5-MeO-DMT) and 4-hy­droxy-*N*,*N*-di­methyl­tryptamine (psilocin) which are known agonists of the serotonin 2A (5-HT_2A_) receptor and elicit a psychedelic response in humans. These and similar compounds have attracted a great deal of inter­est due to their potential for treating conditions including depression (Mertens *et al.*, 2020 ▸), end-of-life distress (Ross *et al.*, 2021 ▸), post-traumatic stress disorder (Varker *et al.*, 2021 ▸), pain (Ramaekers *et al.*, 2021 ▸), and eating disorders (Spriggs *et al.*, 2021 ▸). There are also many synthetic tertiary tryptamines used as pharmaceuticals including the triptans, which have long been used for the treatment of migraine headaches by activating the serotonin 1D (5-HT_1D_) receptor (Goadsby & Holland, 2018 ▸). The biological impact of primary and tertiary tryptamines has been recognized for a long time and continues to be studied in great detail today.

Much less studied are the secondary tryptamines, *i.e.* the mono­alkyl­tryptamines; many of these compounds have been observed as natural products in plants. One study suggests that mono­alkyl­tryptamines are generally less toxic than their di­alkyl­tryptamine counterparts (Brimblecombe *et al.*, 1964 ▸). For example, the LD_50_ values for *N*-methyl­tryptamine (NMT) and *N*,*N*-di­methyl­tryptamine (DMT) in mice were 78 and 43 mg kg^−1^, respectively. Recent studies have suggested that the psychedelic effects of compounds may not be necessary for the expression of therapeutic effects (Olson, 2021 ▸). Monoalkyl tryptamines like norpsilocin (4-hy­droxy-*N*-methyl­tryptamine) are agonists of 5-HT_2A_ but do not produce head-twitch response (HTR) in mice, which is characteristic of classic psychedelics such as psilocybin and LSD (Sherwood *et al.*, 2020 ▸; Glatfelter *et al.*, 2022*a* ▸). Human studies have found that the compound 5-*tert*-butyl-*N*-methyl­tryptamine is a full agonist of 5-HT_1D_ with a higher binding affinity (K_i_ = 0.45 n*M*) and selectivity five times more potent (EC_50_ = 0.22 n*M*) than the migraine drug naratriptan (EC_50_ = 1.6 nM) (Xu *et al.*, 1999 ▸; Slassi *et al.*, 2000 ▸). These and other data points suggest that mono­alkyl­tryptamines possess characteristics that are conducive to the development of medicines.

Continuing our exploration of mono­alkyl­tryptamines, we present here the first crystal structure of a mono-cyclo­alkyl­tryptamine, *N*-cyclo­hexyl­tryptamine. The compound was synthesized in 1971 *via* the condensation of tryptamine with cyclo­hexa­none followed by reduction with Raney Nickel (Gerecs *et al.*, 1971 ▸). Herein, we report three structures of *N*-cyclo­hexyl­tryptamine compounds, including freebase, bromide and fumarate salts, the later of which represents the first fumarate salt of a mono-cyclo­alkyl­tryptamine.

## 2. Structural commentary

**Figure 1:**
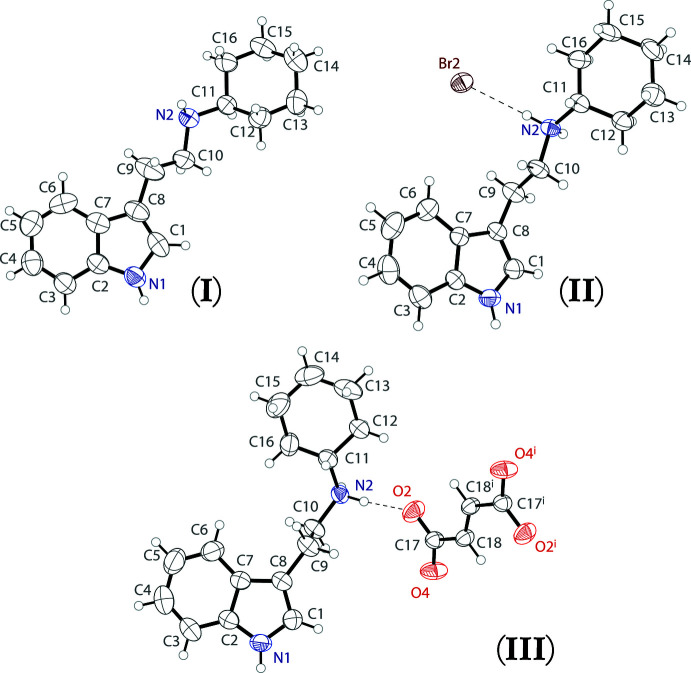
The mol­ecular structures of freebase *N*-cyclo­hexyl­tryptamine (top left), its bromide salt (top right), and its fumarate salt (bottom), showing atomic labeling. Displacement ellipsoids are drawn at the 50% probability level. Hydrogen bonds are shown as dashed lines.

The mol­ecular structure of the freebase of *N*-cyclo­hexyl­tryptamine (**I**) is shown in Fig. 1 ▸ (top left), as well as that of its bromide salt [(**II**), top right], and its fumarate salt [(**III**), bottom]. The asymmetric unit of (**I**) contains one full tryptamine (C_16_H_22_N_2_) mol­ecule. The asymmetric unit of the bromide salt (**II**) contains one *N*-cyclo­hexyl­tryptammonium (C_16_H_23_N_2_^+^) cation and one bromide anion held together with an N2—H2*A*⋯Br1 hydrogen bond. The asymmetric unit of the fumarate salt (**III**) contains one full *N*-cyclo­hexyl­tryptammonium (C_16_H_23_N_2_^+^) cation and one half of a fumarate (C_4_H_2_O_4_^2–^) dianion, with the second half generated by inversion. The two ions are connected in the asymmetric unit through a N2—H2⋯O2 hydrogen bond. The fumarate dianion is near planar, with an r.m.s. deviation from planarity of 0.011 Å. In all three structures, the cyclo­hexyl group is in a chair configuration. Table 1 ▸ lists selected parameters for the three structures.

## 3. Supra­molecular features

**Figure 2:**
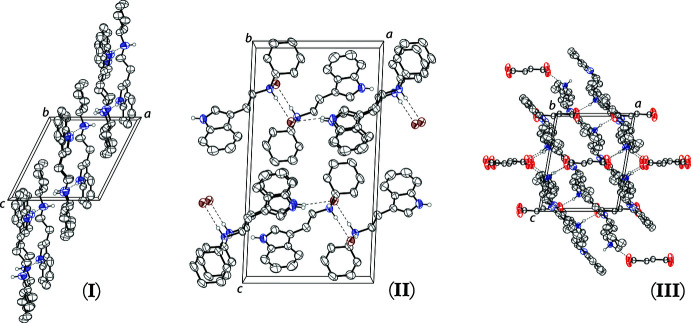
The crystal packing of freebase *N*-cyclo­hexyl­tryptamine (left), its bromide salt (center), and its fumarate salt (right), all shown along the *b*-axis. Hydrogen bonds are shown as dashed lines. H atoms not involved in hydrogen bonding are omitted for clarity.

In the freebase, the tryptamine mol­ecules are held together in infinite chains along [010] by N1—H1⋯N2 hydrogen bonds (Table 2 ▸). In the bromide, the tryptammonium cations and bromide anions are held together in two-dimensional sheets along (001) through a series of N—H⋯Br hydrogen bonds (Table 3 ▸). In the fumarate salt, the tryptammonium cations and fumarate dianions are held together in an infinite three-dimensional framework through a series of N—H⋯O hydrogen bonds. The indole N—H and both ammonium N—H bonds hydrogen bond to oxygen atoms of the fumarate dianions (Table 4 ▸). The packing of *N*-cyclo­hexyl­tryptamine is shown in Fig. 2 ▸ for the freebase (left), the bromide (center) and the fumarate (right).

## 4. Database survey

There are only seven crystal structures of mono­alkyl­tryptamine previously reported. This includes the zwitterionic natural product baeocystin (Naeem, Sherwood *et al.*, 2022 ▸), its metabolite norpsilocin as both its freebase and fumarate (Chadeayne *et al.*, 2020*b* ▸), and its synthetic prodrug 4-acet­oxy-*N*-methyl­tryptamine as a chloride salt (Glatfelter *et al.*, 2022*b* ▸). The remaining three are *N*-methyl­serotonin hydrogen oxalate (Naeem, Anas *et al.*, 2023 ▸), 4-benz­yloxy-*N*-iso­propyl­tryptammonium chloride and 4-hy­droxy-*N*-iso­propyl­tryptamine (Laban *et al.*, 2023 ▸)

There are only four structures of freebase tryptamines known without indole substitution: the natural products tryptamine (Nowell *et al.*, 2002 ▸) and *N*,*N*-di­methyl­tryptamine (Falkenberg, 1972 ▸), as well as *N*-methyl-*N*-propyl­tryptamine (Chadeayne *et al.*, 2019*b* ▸), and 3-[2-(piperidin-1-yl)eth­yl]-1*H*-indole (Sahoo *et al.*, 2020 ▸), while many other tryptamine freebases have been reported including serotonin (Naeem, Chadeayne *et al.*, 2022 ▸).

The crystal structure of only one tryptammonium bromide salt has been presented, that of the natural product *N*,*N*-di­methyl­tryptamine (Falkenberg, 1972 ▸), though numerous chloride salts have been reported (Pham, Belanger *et al.*, 2021 ▸). By contrast, eight bis­(tryptammonium) fumarate structures have been reported recently, including the salts of norpsilocin (Chadeayne *et al.*, 2020*b* ▸), 4-acet­oxy-*N*,*N*-di­allyl­tryptamine (Pham *et al.*, 2021*a* ▸), 5-meth­oxy-*N*,*N*-di­allyl­tryptamine (Pham, Sammeta *et al.*, 2021 ▸), 5-meth­oxy-*N*,*N*-di-*n*-propyl­tryptamine (Pham *et al.*, 2021*c* ▸), 4-hy­droxy-*N*-methyl-*N*-iso­propyl­tryptamine (Chadeayne *et al.*, 2020*a* ▸), 5-meth­oxy-2-methyl-*N*,*N*-di­methyl­tryptamine (Pham *et al.*, 2021*b* ▸), 4-hy­droxy-*N*,*N*-di-*n*-propyl­tryptamine (Chadeayne, Pham *et al.*, 2019 ▸), and 4-acet­oxy-*N*,*N*-di­methyl­tryptamine (Chadeayne *et al.*, 2019*a* ▸).

## 5. Synthesis and crystallization

Crystals of *N*-cyclo­hexyl­tryptammonium bromide (**II**) suitable for X-ray diffraction studies were grown by slow evaporation of an ethanol solution of a commercial sample (ChemBridge).

The bromide salt was converted to freebase *N*-cyclo­hexyl­tryptamine (**I**) by stirring it in a biphasic mixture of di­chloro­ethane and aqueous sodium hydroxide. The organic layer was isolated, washed with brine and dried over sodium sulfate. The solvent was removed *in vacuo* to yield the freebase as a white powder. Crystals suitable for X-ray diffraction were grown by the slow evaporation of an acetone solution.

Freebase *N*-cyclo­hexyl­tryptamine and fumaric acid were dissolved in methanol and heated at reflux for 12 h. The solvent was removed *in vacuo* to yield an off-white powder which was characterized by NMR. Single crystals of (**III**) suitable for X-ray diffraction studies were grown from the slow evaporation of a methanol/water solution. ^1^H NMR (400 MHz, DMSO-*d*_6_): *δ* 7.55 (*d*, *J* = 7.8 Hz, 1H, Ar*H*), 7.35 (*d*, *J* = 8.1 Hz, 1H, Ar*H*), 7.21 (*s*, 1H, Ar*H*), 7.07 (*t*, *J* = 7.5 Hz, 1H, Ar*H*), 6.99 (*t*, *J* = 7.4 Hz, 1H, Ar*H*), 6.43 (*s*, 1H, C*H*), 3.08 (*t*, *J* = 8.3 Hz, 2H, C*H*_2_), 2.99 (*t*, *J* = 8.1 Hz, 2H, C*H*_2_), 2.89 (*m*, 1H, C*H*), 1.98 (*m*, 2H, C*H*_2_), 1.72 (*m*, 2H, C*H*_2_), 1.19 (*m*, 6H, C*H*_2_).

## 6. Refinement

Crystal data, data collection and structure refinement details are summarized in Table 5 ▸. Hydrogen atoms H1 and H2 in the freebase, H1, H2*A* and H2*B* in the bromide salt, and H1, H2*A* and H2*B* in the fumarate salt were found from difference-Fourier maps. These hydrogen atoms were refined isotropically, using DFIX restraints with N—H(indole) distances of 0.87 (1) Å and N—H(amine/ammonium) distances of 0.90 (1) Å. Isotropic displacement parameters were set to 1.2 *U*_eq_ of the parent nitro­gen atoms. All other hydrogen atoms were placed in calculated positions.

## Supplementary Material

Crystal structure: contains datablock(s) I, II, III. DOI: 10.1107/S2056989023006217/tx2070sup1.cif

Structure factors: contains datablock(s) I. DOI: 10.1107/S2056989023006217/tx2070Isup2.hkl

Structure factors: contains datablock(s) II. DOI: 10.1107/S2056989023006217/tx2070IIsup3.hkl

Structure factors: contains datablock(s) III. DOI: 10.1107/S2056989023006217/tx2070IIIsup4.hkl

CCDC references: 2281772, 2281771, 2281770

Additional supporting information:  crystallographic information; 3D view; checkCIF report

## Figures and Tables

**Table 1:** Selected metrical parameters (Å, °) for (**I**)—(**III**)

Compound	indole r.m.s. deviation from planarity	C7—C8—C9—C10	C10—N2—C11
(**I**)	0.007	45.5 (4)	116.6 (3)
(**II**)	0.010	84.2 (5)	114.5 (3)
(**III**)	0.008	−74.77 (19)	117.72 (11)

**Table 2:** Hydrogen-bond geometry (Å, °) for (**I**)

*D*—H⋯*A*	*D*—H	H⋯*A*	*D*⋯*A*	*D*—H⋯*A*
N1—H1⋯N2^i^	0.86 (1)	2.22 (2)	3.069 (4)	167 (3)

Symmetry code: (i) 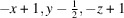 .

**Table 3:** Hydrogen-bond geometry (Å, °) for (**II**)

*D*—H⋯*A*	*D*—H	H⋯*A*	*D*⋯*A*	*D*—H⋯*A*
N2—H2*A*⋯Br1	0.90 (1)	2.41 (1)	3.307 (4)	172 (4)
N1—H1⋯Br1^i^	0.87 (1)	2.68 (3)	3.468 (4)	151 (4)
N2—H2*B*⋯Br1^ii^	0.90 (1)	2.47 (2)	3.340 (3)	163 (4)

Symmetry codes: (i) 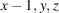 ; (ii) 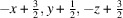 .

**Table 4:** Hydrogen-bond geometry (Å, °) for (**III**)

*D*—H⋯*A*	*D*—H	H⋯*A*	*D*⋯*A*	*D*—H⋯*A*
N2—H2*A*⋯O2	0.91 (1)	1.81 (1)	2.7107 (15)	175 (2)
N1—H1⋯O4^i^	0.88 (1)	1.94 (1)	2.7899 (16)	163 (2)
N2—H2*B*⋯O4^ii^	0.91 (1)	1.87 (1)	2.7632 (16)	167 (2)

Symmetry codes: (i) 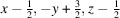 ; (ii) 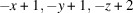 .

**Table 5:** Experimental details

	(**II**)	(**II**)	(**III**)
Crystal data			
Chemical formula	C_16_H_22_N_2_	C_16_H_23_N_2_^+^·Br^−^	C_16_H_23_N_2_^+^·C_2_HO_2_^−^
*M*_r_	242.35	323.27	300.39
Crystal system, space group	Monoclinic, *P*2_1_	Monoclinic, *P*2_1_/*n*	Monoclinic, *P*2_1_/*n*
Temperature (K)	297	297	297
*a*, *b*, *c* (Å)	8.5446 (6), 10.3990 (7), 8.6149 (6)	10.5584 (6), 7.9266 (5), 19.4507 (13)	9.2231 (10), 16.1611 (16), 11.4595 (12)
β (°)	116.784 (2)	92.406 (2)	99.865 (4)
*V* (Å^3^)	683.35 (8)	1626.44 (18)	1682.8 (3)
*Z*	2	4	4
Radiation type	Mo *K*α	Mo *K*α	Mo *K*α
μ (mm^−1^)	0.07	2.52	0.08
Crystal size (mm)	0.35 × 0.24 × 0.2	0.3 × 0.13 × 0.03	0.32 × 0.22 × 0.2

Data collection			
Diffractometer	Bruker D8 Venture CMOS	Bruker D8 Venture CMOS	Bruker D8 Venture CMOS
Absorption correction	Multi-scan (*SADABS*; Krause *et al.*, 2015 ▸)	Multi-scan (*SADABS*; Krause *et al.*, 2015 ▸)	Multi-scan (*SADABS*; Krause *et al.*, 2015 ▸)
*T*_min_, *T*_max_	0.645, 0.745	0.610, 0.745	0.694, 0.745
No. of measured, independent and observed [*I* > 2σ(*I*)] reflections	18695, 2621, 2396	46579, 3320, 2978	20060, 3446, 2803
*R*_int_	0.034	0.037	0.028
(sin θ/λ)_max_ (Å^−1^)	0.613	0.626	0.626

Refinement			
*R*[*F* ^2^ > 2σ(*F* ^2^)], *wR*(*F* ^2^), *S*	0.048, 0.124, 1.04	0.045, 0.109, 1.22	0.043, 0.121, 1.03
No. of reflections	2621	3320	3446
No. of parameters	171	184	211
No. of restraints	3	3	3
H-atom treatment	H atoms treated by a mixture of independent and constrained refinement	H atoms treated by a mixture of independent and constrained refinement	H atoms treated by a mixture of independent and constrained refinement
Δρ_max_, Δρ_min_ (e Å^−3^)	0.26, −0.16	0.58, −0.70	0.22, −0.17
Absolute structure	Flack *x* determined using 1039 quotients [(*I* ^+^)−(*I* ^−^)]/[(*I* ^+^)+(*I* ^−^)] (Parsons et al., 2013 ▸)	–	–
Absolute structure parameter	0.5 (7)	–	–

Computer programs: *APEX4* and *SAINT* (Bruker, 2021 ▸), *SHELXT2014* (Sheldrick, 2015*a* ▸), *SHELXL2018* (Sheldrick, 2015*b* ▸), *OLEX2* (Dolomanov *et al.*, 2009 ▸), and *publCIF* (Westrip, 2010 ▸).

## supplementary crystallographic information

### N-[2-(1H-Indol-3-yl)ethyl]cyclohexanamine (I) Crystal data

**Table:**

C_16_H_22_N_2_	*F*(000) = 264
*M**_r_* = 242.35	*D*_x_ = 1.178 Mg m^−^^3^
Monoclinic, *P*2_1_	Mo *K*α radiation, λ = 0.71073 Å
*a* = 8.5446 (6) Å	Cell parameters from 8705 reflections
*b* = 10.3990 (7) Å	θ = 2.7–25.8°
*c* = 8.6149 (6) Å	µ = 0.07 mm^−^^1^
β = 116.784 (2)°	*T* = 297 K
*V* = 683.35 (8) Å^3^	Block, colourless
*Z* = 2	0.35 × 0.24 × 0.2 mm

### N-[2-(1H-Indol-3-yl)ethyl]cyclohexanamine (I) Data collection

**Table:**

Bruker D8 Venture CMOS diffractometer	2396 reflections with *I* > 2σ(*I*)
φ and ω scans	*R*_int_ = 0.034
Absorption correction: multi-scan (SADABS; Krause *et al.*, 2015)	θ_max_ = 25.8°, θ_min_ = 3.3°
*T*_min_ = 0.645, *T*_max_ = 0.745	*h* = −10→10
18695 measured reflections	*k* = −12→12
2621 independent reflections	*l* = −10→10

### N-[2-(1H-Indol-3-yl)ethyl]cyclohexanamine (I) Refinement

**Table:**

Refinement on *F*^2^	Hydrogen site location: mixed
Least-squares matrix: full	H atoms treated by a mixture of independent and constrained refinement
*R*[*F*^2^ > 2σ(*F*^2^)] = 0.048	*w* = 1/[σ^2^(*F*_o_^2^) + (0.0573*P*)^2^ + 0.1584*P*] where *P* = (*F*_o_^2^ + 2*F*_c_^2^)/3
*wR*(*F*^2^) = 0.124	(Δ/σ)_max_ < 0.001
*S* = 1.04	Δρ_max_ = 0.26 e Å^−^^3^
2621 reflections	Δρ_min_ = −0.16 e Å^−^^3^
171 parameters	Absolute structure: Flack *x* determined using 1039 quotients [(*I*^+^)-(*I*^-^)]/[(*I*^+^)+(*I*^-^)] (Parsons et al., 2013)
3 restraints	Absolute structure parameter: 0.5 (7)

### N-[2-(1H-Indol-3-yl)ethyl]cyclohexanamine (I) Special details

**Table:**

Geometry. All esds (except the esd in the dihedral angle between two l.s. planes) are estimated using the full covariance matrix. The cell esds are taken into account individually in the estimation of esds in distances, angles and torsion angles; correlations between esds in cell parameters are only used when they are defined by crystal symmetry. An approximate (isotropic) treatment of cell esds is used for estimating esds involving l.s. planes.

### N-[2-(1H-Indol-3-yl)ethyl]cyclohexanamine (I) Fractional atomic coordinates and isotropic or equivalent isotropic displacement parameters (Å^2^)

**Table:**

	*x*	*y*	*z*	*U*_iso_*/*U*_eq_	
N1	0.6840 (4)	0.3201 (3)	0.7683 (3)	0.0574 (6)	
N2	0.4242 (4)	0.5812 (3)	0.0943 (3)	0.0632 (7)	
C1	0.6257 (4)	0.3382 (4)	0.5918 (4)	0.0653 (9)	
H1A	0.570884	0.275362	0.507790	0.078*	
C2	0.7549 (4)	0.4345 (3)	0.8487 (4)	0.0512 (7)	
C3	0.8321 (4)	0.4672 (3)	1.0235 (4)	0.0608 (8)	
H3	0.840170	0.407744	1.107439	0.073*	
C4	0.8964 (5)	0.5900 (4)	1.0695 (5)	0.0692 (9)	
H4	0.950320	0.612645	1.186509	0.083*	
C5	0.8830 (5)	0.6805 (4)	0.9467 (5)	0.0709 (9)	
H5	0.926088	0.763121	0.981803	0.085*	
C6	0.8069 (4)	0.6495 (3)	0.7743 (5)	0.0660 (9)	
H6	0.797958	0.710952	0.692294	0.079*	
C7	0.7419 (4)	0.5244 (3)	0.7208 (4)	0.0539 (7)	
C8	0.6589 (4)	0.4598 (4)	0.5573 (4)	0.0619 (8)	
C9	0.6235 (5)	0.5204 (5)	0.3860 (4)	0.0855 (13)	
H9A	0.642061	0.612333	0.403785	0.103*	
H9B	0.709578	0.488038	0.351364	0.103*	
C10	0.4504 (5)	0.4997 (4)	0.2431 (4)	0.0721 (11)	
H10A	0.361947	0.519546	0.280822	0.086*	
H10B	0.437557	0.410028	0.208452	0.086*	
C11	0.2920 (4)	0.5382 (3)	−0.0766 (3)	0.0492 (6)	
H11	0.177680	0.540657	−0.075896	0.059*	
C12	0.3181 (4)	0.4030 (3)	−0.1291 (4)	0.0596 (8)	
H12A	0.431796	0.397430	−0.128156	0.072*	
H12B	0.315875	0.341579	−0.045337	0.072*	
C13	0.1762 (6)	0.3695 (4)	−0.3085 (5)	0.0757 (10)	
H13A	0.196778	0.283691	−0.339441	0.091*	
H13B	0.063234	0.369608	−0.307234	0.091*	
C14	0.1723 (5)	0.4639 (5)	−0.4431 (5)	0.0786 (11)	
H14A	0.281049	0.458099	−0.452537	0.094*	
H14B	0.076703	0.442457	−0.555435	0.094*	
C15	0.1490 (5)	0.5982 (4)	−0.3946 (4)	0.0705 (9)	
H15A	0.033473	0.606436	−0.400188	0.085*	
H15B	0.155915	0.657786	−0.477959	0.085*	
C16	0.2870 (5)	0.6337 (3)	−0.2129 (4)	0.0657 (9)	
H16A	0.262024	0.718893	−0.183813	0.079*	
H16B	0.401106	0.636367	−0.211442	0.079*	
H2	0.524 (3)	0.594 (5)	0.083 (5)	0.091 (14)*	
H1	0.672 (5)	0.249 (2)	0.813 (4)	0.067 (11)*	

### N-[2-(1H-Indol-3-yl)ethyl]cyclohexanamine (I) Atomic displacement parameters (Å^2^)

**Table:**

	*U*^11^	*U*^22^	*U*^33^	*U*^12^	*U*^13^	*U*^23^
N1	0.0606 (15)	0.0598 (16)	0.0454 (13)	0.0075 (12)	0.0183 (11)	0.0039 (11)
N2	0.0709 (17)	0.0612 (17)	0.0445 (14)	−0.0103 (14)	0.0146 (12)	0.0061 (12)
C1	0.0541 (17)	0.092 (3)	0.0430 (16)	0.0035 (17)	0.0155 (13)	−0.0069 (17)
C2	0.0454 (14)	0.0590 (19)	0.0470 (15)	0.0150 (12)	0.0188 (12)	0.0066 (12)
C3	0.0663 (19)	0.064 (2)	0.0512 (16)	0.0055 (16)	0.0256 (15)	0.0008 (15)
C4	0.070 (2)	0.074 (2)	0.066 (2)	−0.0022 (18)	0.0326 (17)	−0.0135 (19)
C5	0.069 (2)	0.066 (2)	0.085 (3)	−0.0005 (17)	0.041 (2)	−0.0092 (19)
C6	0.0566 (17)	0.061 (2)	0.092 (3)	0.0139 (15)	0.0436 (18)	0.0245 (18)
C7	0.0422 (14)	0.0677 (19)	0.0545 (16)	0.0148 (14)	0.0243 (13)	0.0098 (15)
C8	0.0475 (16)	0.087 (2)	0.0490 (17)	0.0113 (16)	0.0200 (14)	0.0142 (16)
C9	0.062 (2)	0.136 (4)	0.0550 (19)	0.008 (2)	0.0229 (16)	0.027 (2)
C10	0.079 (2)	0.073 (2)	0.0464 (17)	−0.0146 (17)	0.0123 (16)	0.0137 (15)
C11	0.0474 (14)	0.0523 (16)	0.0432 (14)	0.0020 (12)	0.0162 (11)	0.0029 (12)
C12	0.0606 (17)	0.0540 (18)	0.0619 (18)	0.0051 (15)	0.0255 (15)	0.0053 (15)
C13	0.088 (3)	0.064 (2)	0.068 (2)	−0.0063 (18)	0.0290 (19)	−0.0126 (17)
C14	0.085 (3)	0.097 (3)	0.0528 (19)	0.001 (2)	0.0302 (18)	−0.0077 (19)
C15	0.071 (2)	0.088 (3)	0.0477 (17)	0.0130 (19)	0.0224 (15)	0.0168 (17)
C16	0.084 (2)	0.0551 (19)	0.0510 (18)	0.0032 (16)	0.0247 (17)	0.0092 (14)

### N-[2-(1H-Indol-3-yl)ethyl]cyclohexanamine (I) Geometric parameters (Å, º)

**Table:**

N1—C1	1.383 (4)	C9—H9B	0.9700
N1—C2	1.372 (4)	C9—C10	1.452 (5)
N1—H1	0.864 (14)	C10—H10A	0.9700
N2—C10	1.467 (4)	C10—H10B	0.9700
N2—C11	1.464 (4)	C11—H11	0.9800
N2—H2	0.912 (14)	C11—C12	1.523 (4)
C1—H1A	0.9300	C11—C16	1.524 (4)
C1—C8	1.357 (5)	C12—H12A	0.9700
C2—C3	1.388 (4)	C12—H12B	0.9700
C2—C7	1.410 (4)	C12—C13	1.514 (5)
C3—H3	0.9300	C13—H13A	0.9700
C3—C4	1.375 (5)	C13—H13B	0.9700
C4—H4	0.9300	C13—C14	1.508 (6)
C4—C5	1.382 (5)	C14—H14A	0.9700
C5—H5	0.9300	C14—H14B	0.9700
C5—C6	1.364 (5)	C14—C15	1.497 (6)
C6—H6	0.9300	C15—H15A	0.9700
C6—C7	1.408 (5)	C15—H15B	0.9700
C7—C8	1.428 (5)	C15—C16	1.521 (5)
C8—C9	1.505 (4)	C16—H16A	0.9700
C9—H9A	0.9700	C16—H16B	0.9700
			
C1—N1—H1	124 (3)	C9—C10—H10A	109.5
C2—N1—C1	107.2 (3)	C9—C10—H10B	109.5
C2—N1—H1	129 (2)	H10A—C10—H10B	108.1
C10—N2—H2	113 (3)	N2—C11—H11	107.8
C11—N2—C10	116.6 (3)	N2—C11—C12	115.5 (3)
C11—N2—H2	106 (2)	N2—C11—C16	108.5 (2)
N1—C1—H1A	124.5	C12—C11—H11	107.8
C8—C1—N1	111.1 (3)	C12—C11—C16	109.3 (2)
C8—C1—H1A	124.5	C16—C11—H11	107.8
N1—C2—C3	130.3 (3)	C11—C12—H12A	109.4
N1—C2—C7	108.6 (3)	C11—C12—H12B	109.4
C3—C2—C7	121.1 (3)	H12A—C12—H12B	108.0
C2—C3—H3	120.9	C13—C12—C11	111.0 (3)
C4—C3—C2	118.3 (3)	C13—C12—H12A	109.4
C4—C3—H3	120.9	C13—C12—H12B	109.4
C3—C4—H4	119.1	C12—C13—H13A	109.3
C3—C4—C5	121.8 (4)	C12—C13—H13B	109.3
C5—C4—H4	119.1	H13A—C13—H13B	108.0
C4—C5—H5	119.8	C14—C13—C12	111.4 (3)
C6—C5—C4	120.5 (4)	C14—C13—H13A	109.3
C6—C5—H5	119.8	C14—C13—H13B	109.3
C5—C6—H6	120.0	C13—C14—H14A	109.5
C5—C6—C7	119.9 (3)	C13—C14—H14B	109.5
C7—C6—H6	120.0	H14A—C14—H14B	108.1
C2—C7—C8	106.7 (3)	C15—C14—C13	110.6 (3)
C6—C7—C2	118.4 (3)	C15—C14—H14A	109.5
C6—C7—C8	134.9 (3)	C15—C14—H14B	109.5
C1—C8—C7	106.4 (3)	C14—C15—H15A	109.2
C1—C8—C9	129.4 (4)	C14—C15—H15B	109.2
C7—C8—C9	124.1 (4)	C14—C15—C16	112.0 (3)
C8—C9—H9A	108.2	H15A—C15—H15B	107.9
C8—C9—H9B	108.2	C16—C15—H15A	109.2
H9A—C9—H9B	107.3	C16—C15—H15B	109.2
C10—C9—C8	116.5 (3)	C11—C16—H16A	109.2
C10—C9—H9A	108.2	C11—C16—H16B	109.2
C10—C9—H9B	108.2	C15—C16—C11	112.0 (3)
N2—C10—H10A	109.5	C15—C16—H16A	109.2
N2—C10—H10B	109.5	C15—C16—H16B	109.2
C9—C10—N2	110.6 (3)	H16A—C16—H16B	107.9
			
N1—C1—C8—C7	−0.6 (4)	C4—C5—C6—C7	−0.2 (5)
N1—C1—C8—C9	178.0 (3)	C5—C6—C7—C2	1.1 (4)
N1—C2—C3—C4	178.7 (3)	C5—C6—C7—C8	−179.2 (3)
N1—C2—C7—C6	179.9 (3)	C6—C7—C8—C1	−179.4 (3)
N1—C2—C7—C8	0.2 (3)	C6—C7—C8—C9	1.9 (5)
N2—C11—C12—C13	−179.1 (3)	C7—C2—C3—C4	−0.2 (4)
N2—C11—C16—C15	−178.4 (3)	C7—C8—C9—C10	−134.5 (4)
C1—N1—C2—C3	−179.5 (3)	C8—C9—C10—N2	170.4 (4)
C1—N1—C2—C7	−0.5 (3)	C10—N2—C11—C12	−56.0 (4)
C1—C8—C9—C10	47.2 (6)	C10—N2—C11—C16	−179.0 (3)
C2—N1—C1—C8	0.7 (4)	C11—N2—C10—C9	156.9 (4)
C2—C3—C4—C5	1.2 (5)	C11—C12—C13—C14	58.1 (4)
C2—C7—C8—C1	0.2 (3)	C12—C11—C16—C15	54.9 (4)
C2—C7—C8—C9	−178.4 (3)	C12—C13—C14—C15	−56.4 (4)
C3—C2—C7—C6	−1.0 (4)	C13—C14—C15—C16	54.5 (4)
C3—C2—C7—C8	179.3 (3)	C14—C15—C16—C11	−54.9 (4)
C3—C4—C5—C6	−1.0 (5)	C16—C11—C12—C13	−56.5 (4)

### N-[2-(1H-Indol-3-yl)ethyl]cyclohexanamine (I) Hydrogen-bond geometry (Å, º)

**Table:**

*D*—H···*A*	*D*—H	H···*A*	*D*···*A*	*D*—H···*A*
N1—H1···N2^i^	0.86 (1)	2.22 (2)	3.069 (4)	167 (3)

Symmetry code: (i) −*x*+1, *y*−1/2, −*z*+1.

### N-[2-(1H-Indol-3-yl)ethyl]cyclohexanaminium bromide (II) Crystal data

**Table:**

C_16_H_23_N_2_^+^·Br^−^	*F*(000) = 672
*M**_r_* = 323.27	*D*_x_ = 1.320 Mg m^−^^3^
Monoclinic, *P*2_1_/*n*	Mo *K*α radiation, λ = 0.71073 Å
*a* = 10.5584 (6) Å	Cell parameters from 9846 reflections
*b* = 7.9266 (5) Å	θ = 2.8–26.3°
*c* = 19.4507 (13) Å	µ = 2.52 mm^−^^1^
β = 92.406 (2)°	*T* = 297 K
*V* = 1626.44 (18) Å^3^	Block, colourless
*Z* = 4	0.3 × 0.13 × 0.03 mm

### N-[2-(1H-Indol-3-yl)ethyl]cyclohexanaminium bromide (II) Data collection

**Table:**

Bruker D8 Venture CMOS diffractometer	2978 reflections with *I* > 2σ(*I*)
φ and ω scans	*R*_int_ = 0.037
Absorption correction: multi-scan (SADABS; Krause *et al.*, 2015)	θ_max_ = 26.4°, θ_min_ = 3.2°
*T*_min_ = 0.610, *T*_max_ = 0.745	*h* = −13→13
46579 measured reflections	*k* = −9→9
3320 independent reflections	*l* = −24→24

### N-[2-(1H-Indol-3-yl)ethyl]cyclohexanaminium bromide (II) Refinement

**Table:**

Refinement on *F*^2^	3 restraints
Least-squares matrix: full	Hydrogen site location: mixed
*R*[*F*^2^ > 2σ(*F*^2^)] = 0.045	H atoms treated by a mixture of independent and constrained refinement
*wR*(*F*^2^) = 0.109	*w* = 1/[σ^2^(*F*_o_^2^) + 4.8013*P*] where *P* = (*F*_o_^2^ + 2*F*_c_^2^)/3
*S* = 1.22	(Δ/σ)_max_ = 0.001
3320 reflections	Δρ_max_ = 0.58 e Å^−^^3^
184 parameters	Δρ_min_ = −0.70 e Å^−^^3^

### N-[2-(1H-Indol-3-yl)ethyl]cyclohexanaminium bromide (II) Special details

**Table:**

Geometry. All esds (except the esd in the dihedral angle between two l.s. planes) are estimated using the full covariance matrix. The cell esds are taken into account individually in the estimation of esds in distances, angles and torsion angles; correlations between esds in cell parameters are only used when they are defined by crystal symmetry. An approximate (isotropic) treatment of cell esds is used for estimating esds involving l.s. planes.

### N-[2-(1H-Indol-3-yl)ethyl]cyclohexanaminium bromide (II) Fractional atomic coordinates and isotropic or equivalent isotropic displacement parameters (Å^2^)

**Table:**

	*x*	*y*	*z*	*U*_iso_*/*U*_eq_	
Br1	0.81840 (4)	0.64728 (5)	0.83341 (2)	0.04595 (14)	
N1	0.1405 (3)	0.7180 (5)	0.8197 (2)	0.0510 (9)	
N2	0.6414 (3)	0.7350 (4)	0.69368 (18)	0.0362 (7)	
C1	0.2277 (4)	0.7985 (6)	0.7822 (2)	0.0514 (11)	
H1A	0.208468	0.877881	0.748124	0.062*	
C2	0.2018 (4)	0.6091 (5)	0.8650 (2)	0.0397 (9)	
C3	0.1549 (5)	0.5037 (6)	0.9141 (2)	0.0542 (12)	
H3	0.068295	0.495965	0.920528	0.065*	
C4	0.2401 (6)	0.4109 (7)	0.9529 (3)	0.0680 (15)	
H4	0.211162	0.339725	0.986910	0.082*	
C5	0.3704 (6)	0.4212 (7)	0.9425 (3)	0.0709 (15)	
H5	0.425959	0.354368	0.968979	0.085*	
C6	0.4180 (5)	0.5268 (6)	0.8944 (2)	0.0544 (11)	
H6	0.504805	0.534207	0.888792	0.065*	
C7	0.3330 (4)	0.6233 (5)	0.85403 (19)	0.0364 (8)	
C8	0.3467 (4)	0.7472 (5)	0.8011 (2)	0.0391 (9)	
C9	0.4690 (4)	0.8051 (6)	0.7726 (2)	0.0461 (10)	
H9A	0.533883	0.809465	0.809400	0.055*	
H9B	0.458286	0.918033	0.754113	0.055*	
C10	0.5116 (4)	0.6891 (6)	0.7169 (2)	0.0443 (10)	
H10A	0.451375	0.694700	0.677896	0.053*	
H10B	0.512820	0.574023	0.733848	0.053*	
C11	0.6844 (4)	0.6344 (5)	0.6336 (2)	0.0398 (9)	
H11	0.666538	0.515102	0.642099	0.048*	
C12	0.6154 (4)	0.6853 (6)	0.5674 (2)	0.0481 (11)	
H12A	0.630562	0.803842	0.558442	0.058*	
H12B	0.524985	0.669392	0.571593	0.058*	
C13	0.6611 (5)	0.5796 (7)	0.5079 (3)	0.0591 (13)	
H13A	0.639096	0.462249	0.515003	0.071*	
H13B	0.618863	0.616791	0.465273	0.071*	
C14	0.8031 (5)	0.5951 (7)	0.5020 (2)	0.0616 (14)	
H14A	0.830564	0.521421	0.465755	0.074*	
H14B	0.824146	0.710124	0.489862	0.074*	
C15	0.8720 (5)	0.5486 (8)	0.5691 (3)	0.0676 (15)	
H15A	0.962363	0.565437	0.564858	0.081*	
H15B	0.857931	0.430226	0.578863	0.081*	
C16	0.8262 (4)	0.6553 (7)	0.6284 (2)	0.0559 (12)	
H16A	0.868998	0.620155	0.671109	0.067*	
H16B	0.846249	0.772986	0.620616	0.067*	
H2A	0.692 (3)	0.722 (6)	0.7316 (13)	0.045 (12)*	
H1	0.0593 (14)	0.738 (6)	0.815 (2)	0.058 (14)*	
H2B	0.636 (4)	0.844 (2)	0.681 (2)	0.045 (12)*	

### N-[2-(1H-Indol-3-yl)ethyl]cyclohexanaminium bromide (II) Atomic displacement parameters (Å^2^)

**Table:**

	*U*^11^	*U*^22^	*U*^33^	*U*^12^	*U*^13^	*U*^23^
Br1	0.0447 (2)	0.0384 (2)	0.0545 (3)	0.00711 (19)	−0.00029 (17)	−0.0026 (2)
N1	0.0337 (18)	0.062 (2)	0.058 (2)	0.0049 (18)	0.0068 (17)	0.014 (2)
N2	0.0331 (17)	0.0334 (18)	0.0425 (19)	−0.0013 (14)	0.0062 (14)	0.0008 (15)
C1	0.048 (2)	0.057 (3)	0.050 (3)	0.008 (2)	0.009 (2)	0.015 (2)
C2	0.043 (2)	0.037 (2)	0.039 (2)	−0.0032 (17)	0.0058 (17)	−0.0037 (17)
C3	0.058 (3)	0.051 (3)	0.055 (3)	−0.017 (2)	0.012 (2)	0.000 (2)
C4	0.091 (4)	0.053 (3)	0.061 (3)	−0.016 (3)	0.007 (3)	0.019 (3)
C5	0.081 (4)	0.059 (3)	0.071 (4)	0.007 (3)	−0.013 (3)	0.019 (3)
C6	0.053 (3)	0.052 (3)	0.058 (3)	0.003 (2)	−0.003 (2)	0.003 (2)
C7	0.039 (2)	0.034 (2)	0.037 (2)	−0.0008 (16)	0.0027 (16)	−0.0057 (16)
C8	0.039 (2)	0.041 (2)	0.039 (2)	0.0003 (17)	0.0087 (16)	−0.0034 (17)
C9	0.046 (2)	0.044 (2)	0.050 (2)	−0.0042 (19)	0.0143 (19)	−0.004 (2)
C10	0.037 (2)	0.051 (3)	0.047 (2)	−0.0087 (19)	0.0136 (18)	−0.004 (2)
C11	0.045 (2)	0.0282 (19)	0.047 (2)	0.0044 (17)	0.0153 (17)	0.0061 (17)
C12	0.044 (2)	0.052 (3)	0.049 (2)	0.007 (2)	0.0075 (19)	−0.006 (2)
C13	0.064 (3)	0.059 (3)	0.054 (3)	0.004 (3)	0.009 (2)	−0.011 (2)
C14	0.064 (3)	0.073 (4)	0.050 (3)	0.011 (3)	0.026 (2)	0.001 (2)
C15	0.051 (3)	0.084 (4)	0.070 (3)	0.021 (3)	0.029 (2)	0.005 (3)
C16	0.040 (2)	0.068 (3)	0.060 (3)	0.011 (2)	0.012 (2)	0.010 (3)

### N-[2-(1H-Indol-3-yl)ethyl]cyclohexanaminium bromide (II) Geometric parameters (Å, º)

**Table:**

N1—C1	1.358 (6)	C9—H9B	0.9700
N1—C2	1.375 (5)	C9—C10	1.504 (6)
N1—H1	0.873 (10)	C10—H10A	0.9700
N2—C10	1.506 (5)	C10—H10B	0.9700
N2—C11	1.501 (5)	C11—H11	0.9800
N2—H2A	0.898 (10)	C11—C12	1.506 (6)
N2—H2B	0.900 (10)	C11—C16	1.515 (6)
C1—H1A	0.9300	C12—H12A	0.9700
C1—C8	1.357 (6)	C12—H12B	0.9700
C2—C3	1.376 (6)	C12—C13	1.525 (6)
C2—C7	1.415 (5)	C13—H13A	0.9700
C3—H3	0.9300	C13—H13B	0.9700
C3—C4	1.366 (7)	C13—C14	1.513 (7)
C4—H4	0.9300	C14—H14A	0.9700
C4—C5	1.402 (8)	C14—H14B	0.9700
C5—H5	0.9300	C14—C15	1.513 (7)
C5—C6	1.366 (7)	C15—H15A	0.9700
C6—H6	0.9300	C15—H15B	0.9700
C6—C7	1.396 (6)	C15—C16	1.525 (7)
C7—C8	1.434 (6)	C16—H16A	0.9700
C8—C9	1.499 (5)	C16—H16B	0.9700
C9—H9A	0.9700		
			
C1—N1—C2	109.2 (4)	C9—C10—N2	111.8 (3)
C1—N1—H1	123 (3)	C9—C10—H10A	109.3
C2—N1—H1	128 (3)	C9—C10—H10B	109.3
C10—N2—H2A	104 (3)	H10A—C10—H10B	107.9
C10—N2—H2B	105 (3)	N2—C11—H11	108.3
C11—N2—C10	114.5 (3)	N2—C11—C12	111.9 (3)
C11—N2—H2A	113 (3)	N2—C11—C16	109.0 (4)
C11—N2—H2B	109 (3)	C12—C11—H11	108.3
H2A—N2—H2B	111 (4)	C12—C11—C16	111.0 (3)
N1—C1—H1A	124.6	C16—C11—H11	108.3
C8—C1—N1	110.7 (4)	C11—C12—H12A	109.6
C8—C1—H1A	124.6	C11—C12—H12B	109.6
N1—C2—C3	130.7 (4)	C11—C12—C13	110.2 (4)
N1—C2—C7	106.9 (3)	H12A—C12—H12B	108.1
C3—C2—C7	122.4 (4)	C13—C12—H12A	109.6
C2—C3—H3	121.2	C13—C12—H12B	109.6
C4—C3—C2	117.6 (5)	C12—C13—H13A	109.4
C4—C3—H3	121.2	C12—C13—H13B	109.4
C3—C4—H4	119.5	H13A—C13—H13B	108.0
C3—C4—C5	121.1 (5)	C14—C13—C12	111.0 (4)
C5—C4—H4	119.5	C14—C13—H13A	109.4
C4—C5—H5	119.1	C14—C13—H13B	109.4
C6—C5—C4	121.8 (5)	C13—C14—H14A	109.4
C6—C5—H5	119.1	C13—C14—H14B	109.4
C5—C6—H6	120.8	H14A—C14—H14B	108.0
C5—C6—C7	118.4 (5)	C15—C14—C13	111.0 (4)
C7—C6—H6	120.8	C15—C14—H14A	109.4
C2—C7—C8	106.9 (3)	C15—C14—H14B	109.4
C6—C7—C2	118.8 (4)	C14—C15—H15A	109.4
C6—C7—C8	134.2 (4)	C14—C15—H15B	109.4
C1—C8—C7	106.3 (4)	C14—C15—C16	111.1 (4)
C1—C8—C9	127.6 (4)	H15A—C15—H15B	108.0
C7—C8—C9	126.1 (4)	C16—C15—H15A	109.4
C8—C9—H9A	109.3	C16—C15—H15B	109.4
C8—C9—H9B	109.3	C11—C16—C15	109.6 (4)
C8—C9—C10	111.6 (3)	C11—C16—H16A	109.7
H9A—C9—H9B	108.0	C11—C16—H16B	109.7
C10—C9—H9A	109.3	C15—C16—H16A	109.7
C10—C9—H9B	109.3	C15—C16—H16B	109.7
N2—C10—H10A	109.3	H16A—C16—H16B	108.2
N2—C10—H10B	109.3		
			
N1—C1—C8—C7	0.8 (5)	C4—C5—C6—C7	−1.5 (8)
N1—C1—C8—C9	−179.9 (4)	C5—C6—C7—C2	0.6 (7)
N1—C2—C3—C4	−178.8 (5)	C5—C6—C7—C8	178.3 (5)
N1—C2—C7—C6	179.1 (4)	C6—C7—C8—C1	−178.9 (5)
N1—C2—C7—C8	0.9 (4)	C6—C7—C8—C9	1.8 (8)
N2—C11—C12—C13	−179.6 (4)	C7—C2—C3—C4	0.0 (7)
N2—C11—C16—C15	177.9 (4)	C7—C8—C9—C10	84.2 (5)
C1—N1—C2—C3	178.5 (5)	C8—C9—C10—N2	−173.5 (4)
C1—N1—C2—C7	−0.4 (5)	C10—N2—C11—C12	73.0 (4)
C1—C8—C9—C10	−95.0 (6)	C10—N2—C11—C16	−163.9 (4)
C2—N1—C1—C8	−0.3 (6)	C11—N2—C10—C9	−174.5 (3)
C2—C3—C4—C5	−0.8 (8)	C11—C12—C13—C14	−56.6 (5)
C2—C7—C8—C1	−1.0 (5)	C12—C11—C16—C15	−58.5 (5)
C2—C7—C8—C9	179.6 (4)	C12—C13—C14—C15	55.5 (6)
C3—C2—C7—C6	0.1 (6)	C13—C14—C15—C16	−56.0 (6)
C3—C2—C7—C8	−178.2 (4)	C14—C15—C16—C11	57.0 (6)
C3—C4—C5—C6	1.6 (9)	C16—C11—C12—C13	58.4 (5)

### N-[2-(1H-Indol-3-yl)ethyl]cyclohexanaminium bromide (II) Hydrogen-bond geometry (Å, º)

**Table:**

*D*—H···*A*	*D*—H	H···*A*	*D*···*A*	*D*—H···*A*
N2—H2*A*···Br1	0.90 (1)	2.41 (1)	3.307 (4)	172 (4)
N1—H1···Br1^i^	0.87 (1)	2.68 (3)	3.468 (4)	151 (4)
N2—H2*B*···Br1^ii^	0.90 (1)	2.47 (2)	3.340 (3)	163 (4)

Symmetry codes: (i) *x*−1, *y*, *z*; (ii) −*x*+3/2, *y*+1/2, −*z*+3/2.

### Bis{N-[2-(1H-indol-3-yl)ethyl]cyclohexanaminium} (2E)-but-2-enedioate (III) Crystal data

**Table:**

C_16_H_23_N_2_^+^·C_2_HO_2_^−^	*F*(000) = 648
*M**_r_* = 300.39	*D*_x_ = 1.186 Mg m^−^^3^
Monoclinic, *P*2_1_/*n*	Mo *K*α radiation, λ = 0.71073 Å
*a* = 9.2231 (10) Å	Cell parameters from 7849 reflections
*b* = 16.1611 (16) Å	θ = 2.6–26.3°
*c* = 11.4595 (12) Å	µ = 0.08 mm^−^^1^
β = 99.865 (4)°	*T* = 297 K
*V* = 1682.8 (3) Å^3^	Block, bronze
*Z* = 4	0.32 × 0.22 × 0.2 mm

### Bis{N-[2-(1H-indol-3-yl)ethyl]cyclohexanaminium} (2E)-but-2-enedioate (III) Data collection

**Table:**

Bruker D8 Venture CMOS diffractometer	2803 reflections with *I* > 2σ(*I*)
φ and ω scans	*R*_int_ = 0.028
Absorption correction: multi-scan (SADABS; Krause *et al.*, 2015)	θ_max_ = 26.4°, θ_min_ = 2.6°
*T*_min_ = 0.694, *T*_max_ = 0.745	*h* = −11→11
20060 measured reflections	*k* = −20→20
3446 independent reflections	*l* = −14→14

### Bis{N-[2-(1H-indol-3-yl)ethyl]cyclohexanaminium} (2E)-but-2-enedioate (III) Refinement

**Table:**

Refinement on *F*^2^	3 restraints
Least-squares matrix: full	Hydrogen site location: mixed
*R*[*F*^2^ > 2σ(*F*^2^)] = 0.043	H atoms treated by a mixture of independent and constrained refinement
*wR*(*F*^2^) = 0.121	*w* = 1/[σ^2^(*F*_o_^2^) + (0.057*P*)^2^ + 0.4152*P*] where *P* = (*F*_o_^2^ + 2*F*_c_^2^)/3
*S* = 1.03	(Δ/σ)_max_ < 0.001
3446 reflections	Δρ_max_ = 0.22 e Å^−^^3^
211 parameters	Δρ_min_ = −0.17 e Å^−^^3^

### Bis{N-[2-(1H-indol-3-yl)ethyl]cyclohexanaminium} (2E)-but-2-enedioate (III) Special details

**Table:**

Geometry. All esds (except the esd in the dihedral angle between two l.s. planes) are estimated using the full covariance matrix. The cell esds are taken into account individually in the estimation of esds in distances, angles and torsion angles; correlations between esds in cell parameters are only used when they are defined by crystal symmetry. An approximate (isotropic) treatment of cell esds is used for estimating esds involving l.s. planes.

### Bis{N-[2-(1H-indol-3-yl)ethyl]cyclohexanaminium} (2E)-but-2-enedioate (III) Fractional atomic coordinates and isotropic or equivalent isotropic displacement parameters (Å^2^)

**Table:**

	*x*	*y*	*z*	*U*_iso_*/*U*_eq_	
O2	0.72753 (11)	0.47420 (7)	0.97368 (12)	0.0632 (3)	
O4	0.74997 (12)	0.60453 (6)	1.03308 (11)	0.0600 (3)	
N1	0.33759 (16)	0.73002 (8)	0.54717 (12)	0.0530 (3)	
N2	0.46996 (12)	0.42877 (7)	0.83748 (10)	0.0402 (3)	
C1	0.43744 (18)	0.68408 (10)	0.62215 (13)	0.0522 (4)	
H1A	0.513836	0.706133	0.676833	0.063*	
C2	0.24236 (16)	0.67738 (9)	0.48012 (12)	0.0444 (3)	
C3	0.12270 (18)	0.69461 (11)	0.39157 (14)	0.0589 (4)	
H3	0.095302	0.748764	0.371017	0.071*	
C4	0.0471 (2)	0.62879 (15)	0.33603 (17)	0.0753 (5)	
H4	−0.032377	0.638517	0.275820	0.090*	
C5	0.0863 (2)	0.54759 (14)	0.36747 (19)	0.0780 (6)	
H5	0.032566	0.504233	0.327913	0.094*	
C6	0.20302 (19)	0.53036 (10)	0.45603 (16)	0.0609 (4)	
H6	0.227496	0.475901	0.477033	0.073*	
C7	0.28409 (15)	0.59569 (8)	0.51382 (12)	0.0426 (3)	
C8	0.41022 (16)	0.60185 (9)	0.60614 (12)	0.0452 (3)	
C9	0.48890 (17)	0.53145 (10)	0.67518 (14)	0.0523 (4)	
H9A	0.584225	0.550063	0.715932	0.063*	
H9B	0.504732	0.487268	0.621464	0.063*	
C10	0.39908 (16)	0.49921 (9)	0.76478 (13)	0.0475 (3)	
H10A	0.382929	0.544073	0.817299	0.057*	
H10B	0.303662	0.481452	0.723096	0.057*	
C11	0.50069 (15)	0.35126 (8)	0.77365 (12)	0.0423 (3)	
H11	0.565930	0.364743	0.717251	0.051*	
C12	0.57867 (19)	0.29119 (10)	0.86470 (15)	0.0577 (4)	
H12A	0.519809	0.282701	0.926173	0.069*	
H12B	0.672434	0.314467	0.901290	0.069*	
C13	0.6045 (2)	0.20810 (11)	0.80791 (19)	0.0785 (6)	
H13A	0.671411	0.215725	0.752071	0.094*	
H13B	0.649859	0.169933	0.868687	0.094*	
C14	0.4613 (3)	0.17171 (11)	0.74422 (18)	0.0784 (6)	
H14A	0.480951	0.120240	0.706292	0.094*	
H14B	0.397404	0.159654	0.801073	0.094*	
C15	0.3856 (3)	0.23094 (13)	0.65269 (16)	0.0783 (6)	
H15A	0.291923	0.207658	0.615817	0.094*	
H15B	0.445353	0.238438	0.591619	0.094*	
C16	0.35933 (18)	0.31489 (11)	0.70660 (14)	0.0584 (4)	
H16A	0.317379	0.352639	0.644040	0.070*	
H16B	0.289125	0.308535	0.760051	0.070*	
C17	0.80163 (14)	0.53717 (8)	1.00368 (12)	0.0397 (3)	
C18	0.96469 (14)	0.53303 (8)	1.01170 (12)	0.0401 (3)	
H18	1.019151	0.580476	1.034817	0.048*	
H2A	0.5558 (13)	0.4470 (10)	0.8805 (13)	0.061 (5)*	
H1	0.330 (2)	0.7840 (6)	0.5455 (17)	0.075 (6)*	
H2B	0.4089 (16)	0.4143 (10)	0.8881 (12)	0.057 (5)*	

### Bis{N-[2-(1H-indol-3-yl)ethyl]cyclohexanaminium} (2E)-but-2-enedioate (III) Atomic displacement parameters (Å^2^)

**Table:**

	*U*^11^	*U*^22^	*U*^33^	*U*^12^	*U*^13^	*U*^23^
O2	0.0387 (6)	0.0534 (6)	0.0948 (9)	−0.0079 (5)	0.0038 (5)	−0.0168 (6)
O4	0.0520 (6)	0.0374 (5)	0.0972 (9)	0.0050 (4)	0.0316 (6)	−0.0031 (5)
N1	0.0706 (9)	0.0364 (6)	0.0517 (7)	0.0033 (6)	0.0096 (6)	0.0033 (5)
N2	0.0349 (6)	0.0417 (6)	0.0432 (6)	0.0028 (5)	0.0045 (5)	0.0042 (5)
C1	0.0589 (9)	0.0501 (8)	0.0457 (8)	−0.0022 (7)	0.0033 (7)	0.0005 (7)
C2	0.0508 (8)	0.0445 (7)	0.0403 (7)	0.0063 (6)	0.0143 (6)	0.0038 (6)
C3	0.0601 (10)	0.0671 (10)	0.0496 (8)	0.0182 (8)	0.0094 (7)	0.0116 (8)
C4	0.0550 (10)	0.1013 (15)	0.0642 (11)	0.0070 (10)	−0.0047 (8)	0.0026 (11)
C5	0.0691 (12)	0.0806 (14)	0.0785 (13)	−0.0142 (10)	−0.0037 (10)	−0.0156 (11)
C6	0.0632 (10)	0.0485 (9)	0.0706 (10)	−0.0031 (7)	0.0106 (8)	−0.0066 (8)
C7	0.0462 (7)	0.0408 (7)	0.0430 (7)	0.0046 (6)	0.0140 (6)	0.0013 (6)
C8	0.0489 (8)	0.0438 (7)	0.0443 (7)	0.0069 (6)	0.0118 (6)	0.0055 (6)
C9	0.0489 (8)	0.0535 (9)	0.0556 (8)	0.0119 (7)	0.0125 (7)	0.0126 (7)
C10	0.0447 (8)	0.0459 (8)	0.0528 (8)	0.0134 (6)	0.0107 (6)	0.0094 (6)
C11	0.0401 (7)	0.0426 (7)	0.0452 (7)	0.0052 (6)	0.0100 (6)	0.0034 (6)
C12	0.0577 (9)	0.0463 (8)	0.0640 (9)	0.0070 (7)	−0.0046 (7)	0.0086 (7)
C13	0.0968 (15)	0.0521 (10)	0.0886 (13)	0.0254 (10)	0.0213 (12)	0.0133 (10)
C14	0.1227 (18)	0.0475 (10)	0.0713 (12)	−0.0069 (10)	0.0346 (12)	−0.0107 (9)
C15	0.1055 (16)	0.0739 (13)	0.0546 (10)	−0.0116 (11)	0.0112 (10)	−0.0191 (9)
C16	0.0568 (9)	0.0642 (10)	0.0499 (8)	0.0003 (8)	−0.0026 (7)	−0.0062 (7)
C17	0.0356 (6)	0.0357 (7)	0.0482 (7)	0.0013 (5)	0.0082 (5)	0.0001 (5)
C18	0.0363 (7)	0.0346 (6)	0.0493 (7)	−0.0039 (5)	0.0070 (5)	−0.0044 (5)

### Bis{N-[2-(1H-indol-3-yl)ethyl]cyclohexanaminium} (2E)-but-2-enedioate (III) Geometric parameters (Å, º)

**Table:**

O2—C17	1.2411 (16)	C9—H9B	0.9700
O4—C17	1.2572 (16)	C9—C10	1.518 (2)
N1—C1	1.366 (2)	C10—H10A	0.9700
N1—C2	1.362 (2)	C10—H10B	0.9700
N1—H1	0.876 (9)	C11—H11	0.9800
N2—C10	1.4935 (17)	C11—C12	1.514 (2)
N2—C11	1.5018 (18)	C11—C16	1.514 (2)
N2—H2A	0.907 (9)	C12—H12A	0.9700
N2—H2B	0.905 (9)	C12—H12B	0.9700
C1—H1A	0.9300	C12—C13	1.529 (2)
C1—C8	1.359 (2)	C13—H13A	0.9700
C2—C3	1.393 (2)	C13—H13B	0.9700
C2—C7	1.4105 (19)	C13—C14	1.514 (3)
C3—H3	0.9300	C14—H14A	0.9700
C3—C4	1.368 (3)	C14—H14B	0.9700
C4—H4	0.9300	C14—C15	1.501 (3)
C4—C5	1.392 (3)	C15—H15A	0.9700
C5—H5	0.9300	C15—H15B	0.9700
C5—C6	1.376 (3)	C15—C16	1.527 (2)
C6—H6	0.9300	C16—H16A	0.9700
C6—C7	1.394 (2)	C16—H16B	0.9700
C7—C8	1.436 (2)	C17—C18	1.4924 (18)
C8—C9	1.5000 (19)	C18—C18^i^	1.302 (3)
C9—H9A	0.9700	C18—H18	0.9300
			
C1—N1—H1	126.9 (13)	H10A—C10—H10B	107.7
C2—N1—C1	108.39 (12)	N2—C11—H11	108.9
C2—N1—H1	124.6 (13)	N2—C11—C12	107.83 (12)
C10—N2—C11	117.72 (11)	N2—C11—C16	110.67 (11)
C10—N2—H2A	108.3 (11)	C12—C11—H11	108.9
C10—N2—H2B	107.1 (11)	C12—C11—C16	111.52 (13)
C11—N2—H2A	108.4 (11)	C16—C11—H11	108.9
C11—N2—H2B	106.6 (11)	C11—C12—H12A	109.4
H2A—N2—H2B	108.5 (15)	C11—C12—H12B	109.4
N1—C1—H1A	124.5	C11—C12—C13	111.11 (14)
C8—C1—N1	110.95 (14)	H12A—C12—H12B	108.0
C8—C1—H1A	124.5	C13—C12—H12A	109.4
N1—C2—C3	129.78 (14)	C13—C12—H12B	109.4
N1—C2—C7	108.12 (12)	C12—C13—H13A	109.4
C3—C2—C7	122.10 (15)	C12—C13—H13B	109.4
C2—C3—H3	121.3	H13A—C13—H13B	108.0
C4—C3—C2	117.42 (16)	C14—C13—C12	111.08 (16)
C4—C3—H3	121.3	C14—C13—H13A	109.4
C3—C4—H4	119.2	C14—C13—H13B	109.4
C3—C4—C5	121.59 (16)	C13—C14—H14A	109.5
C5—C4—H4	119.2	C13—C14—H14B	109.5
C4—C5—H5	119.4	H14A—C14—H14B	108.1
C6—C5—C4	121.16 (18)	C15—C14—C13	110.62 (16)
C6—C5—H5	119.4	C15—C14—H14A	109.5
C5—C6—H6	120.5	C15—C14—H14B	109.5
C5—C6—C7	119.05 (16)	C14—C15—H15A	109.3
C7—C6—H6	120.5	C14—C15—H15B	109.3
C2—C7—C8	106.59 (12)	C14—C15—C16	111.77 (14)
C6—C7—C2	118.68 (14)	H15A—C15—H15B	107.9
C6—C7—C8	134.73 (14)	C16—C15—H15A	109.3
C1—C8—C7	105.96 (12)	C16—C15—H15B	109.3
C1—C8—C9	127.52 (14)	C11—C16—C15	111.59 (15)
C7—C8—C9	126.43 (13)	C11—C16—H16A	109.3
C8—C9—H9A	109.6	C11—C16—H16B	109.3
C8—C9—H9B	109.6	C15—C16—H16A	109.3
C8—C9—C10	110.27 (12)	C15—C16—H16B	109.3
H9A—C9—H9B	108.1	H16A—C16—H16B	108.0
C10—C9—H9A	109.6	O2—C17—O4	124.62 (13)
C10—C9—H9B	109.6	O2—C17—C18	118.79 (12)
N2—C10—C9	113.68 (11)	O4—C17—C18	116.52 (12)
N2—C10—H10A	108.8	C17—C18—H18	118.0
N2—C10—H10B	108.8	C18^i^—C18—C17	123.95 (16)
C9—C10—H10A	108.8	C18^i^—C18—H18	118.0
C9—C10—H10B	108.8		
			
O2—C17—C18—C18^i^	0.5 (3)	C3—C4—C5—C6	0.0 (3)
O4—C17—C18—C18^i^	177.60 (18)	C4—C5—C6—C7	−0.8 (3)
N1—C1—C8—C7	0.23 (17)	C5—C6—C7—C2	0.7 (2)
N1—C1—C8—C9	−176.30 (14)	C5—C6—C7—C8	−178.90 (17)
N1—C2—C3—C4	178.46 (16)	C6—C7—C8—C1	179.25 (17)
N1—C2—C7—C6	−179.33 (14)	C6—C7—C8—C9	−4.2 (3)
N1—C2—C7—C8	0.35 (16)	C7—C2—C3—C4	−1.0 (2)
N2—C11—C12—C13	176.04 (14)	C7—C8—C9—C10	−74.77 (19)
N2—C11—C16—C15	−173.47 (13)	C8—C9—C10—N2	180.00 (12)
C1—N1—C2—C3	−179.77 (15)	C10—N2—C11—C12	177.27 (12)
C1—N1—C2—C7	−0.21 (16)	C10—N2—C11—C16	−60.50 (16)
C1—C8—C9—C10	101.09 (19)	C11—N2—C10—C9	−60.41 (17)
C2—N1—C1—C8	−0.02 (18)	C11—C12—C13—C14	−56.1 (2)
C2—C3—C4—C5	0.9 (3)	C12—C11—C16—C15	−53.43 (19)
C2—C7—C8—C1	−0.35 (16)	C12—C13—C14—C15	56.9 (2)
C2—C7—C8—C9	176.23 (13)	C13—C14—C15—C16	−56.1 (2)
C3—C2—C7—C6	0.3 (2)	C14—C15—C16—C11	54.6 (2)
C3—C2—C7—C8	179.95 (13)	C16—C11—C12—C13	54.34 (19)

Symmetry code: (i) −*x*+2, −*y*+1, −*z*+2.

### Bis{N-[2-(1H-indol-3-yl)ethyl]cyclohexanaminium} (2E)-but-2-enedioate (III) Hydrogen-bond geometry (Å, º)

**Table:**

*D*—H···*A*	*D*—H	H···*A*	*D*···*A*	*D*—H···*A*
N2—H2*A*···O2	0.91 (1)	1.81 (1)	2.7107 (15)	175 (2)
N1—H1···O4^ii^	0.88 (1)	1.94 (1)	2.7899 (16)	163 (2)
N2—H2*B*···O4^iii^	0.91 (1)	1.87 (1)	2.7632 (16)	167 (2)

Symmetry codes: (ii) *x*−1/2, −*y*+3/2, *z*−1/2; (iii) −*x*+1, −*y*+1, −*z*+2.
